# Effect of Stainless Steel Mesh Structural Parameters on the Temperature Field and Joint Tensile-Shear Performance in CF/PC Resistance Welding

**DOI:** 10.3390/polym17212899

**Published:** 2025-10-30

**Authors:** Zhanyi Geng, Shiyuan Wang, Yiwen Li, Sansan Ao, Yang Li

**Affiliations:** School of Materials Science and Engineering, Tianjin University, Tianjin 300354, China; gzy2023@tju.edu.cn (Z.G.); w_sy07@tju.edu.cn (S.W.); lyw1921@tju.edu.cn (Y.L.); ao33@tju.edu.cn (S.A.)

**Keywords:** continuous carbon fiber-reinforced polycarbonate (CCF/PC), resistance welding, numerical simulation, stainless steel heating element

## Abstract

This study employs 304 stainless steel perforated mesh (SS mesh) as the heating element for the resistance welding of continuous carbon fiber-reinforced polycarbonate (CCF/PC) sheets. An electro-thermal coupled finite element model is developed to investigate the effect of SS mesh structural parameters (aperture shape, aperture area, mesh thickness) and clamping distance on the welding temperature field. The model accurately predicts peak temperatures, with errors of 1–4% compared with experiments. Under identical aperture area, the SS mesh with longer effective current path length and smaller effective cross-sectional area has higher resistance. In addition, the resistance increases significantly with decreasing mesh thickness and increasing aperture size. Reducing the clamping distance effectively improves temperature uniformity across the weld zone and mitigates edge overheating. A novel mesh structure—featuring larger aperture in the welding region and smaller aperture in non-welding region, is designed to improve the temperature uniformity and joint quality. Under optimized welding parameters (14 A, 40 s welding/holding, 0.3 MPa), the joint achieves a maximum tensile shear force of 9.851 kN, a 13.1% improvement over conventional uniform-aperture mesh (8.713 kN).

## 1. Introduction

Fiber-reinforced thermoplastic composites (FRTPs) are increasingly challenging the dominance of traditional thermosetting composites across sectors such as aerospace [1,2], automotive [3,4], energy/power [5], and rail transportation [6]. This shift is driven by FRTPs’ superior mechanical properties (high specific strength and modulus, excellent fatigue and impact resistance), high production efficiency, robust environmental adaptability (requiring no special storage conditions, no shelf-life limitations), and advantageous characteristics such as weldability, reparability, and recyclability [7,8,9]. Despite these advantages, joining FRTPs presents significant challenges. Mechanical fastening suffers from stress concentrations, galvanic corrosion, material damage, weight penalty, and higher costs [10]. Adhesive bonding faces limitations including stringent surface preparation requirements, low peel strength, sensitivity to environmental conditions and contamination, and lengthy curing times [11]. In contrast, welding techniques—particularly resistance welding, ultrasonic welding, and induction welding—offer compelling advantages [12]. Induction welding features high welding speed and the capability to join large or complex-shaped components. However, it requires relatively high equipment costs, and interfacial temperature distribution can be sensitive to transient electromagnetic fields and skin effects [13]. Ultrasonic welding offers high efficiency, cleanliness, and high joint strength [14]. However, it still has certain limitations when joining specimens with large thickness or complex structures, and the design or selection of energy directors is also critical [15,16]. Resistance welding has received increasing attention in FRTP joining methods due to its low equipment cost, process simplicity, fast welding speed, and suitability for thick and complex structures [17,18].

Due to the inherent non-conductivity of FRTPs, a heating element should be introduced at the joining interface prior to resistance welding. Joule heating generated by the electrical current passing through this element provides the necessary heat for the welding process. When the interfacial temperature reaches the matrix’s glass transition temperature (*T*_g_) for amorphous polymers or melting temperature (*T*_m_) for semi-crystalline polymers [19], the thermoplastic matrix softens or melts, enhancing the mobility of polymer chains. Under applied pressure, these chains interdiffuse and entangle, forming a reliable joint upon cooling [20].

The heating element significantly influences not only the interfacial temperature distribution and molten resin flow during welding but also the stress distribution and environmental adaptability of the resulting joint [18]. Consequently, designing or selecting an appropriate heating element is critical for ensuring resistance welding quality. Currently, metal mesh and carbon fiber (CF) heating elements are the primary choices for FRTP resistance welding [17]. CF offers advantages including low density, good compatibility with the polymer matrix, and excellent corrosion and fatigue resistance in the welded joint [17]. However, the anisotropic thermoelectric properties of CF increase the difficulty of controlling welding temperature, and the fibers are prone to brittle fracture or oxidative degradation during welding [18]. Although methods such as in situ deposition of carbon nanotubes (CNTs) [21] or edge addition of multi-walled carbon nanotubes (MWCNTs) [22] have been proposed to enhance CF performance, these approaches increase joining costs. In contrast, metal meshes, particularly stainless steel (SS) mesh, are widely employed in FRTP resistance welding due to their low cost, excellent electrical and thermal conductivity, strong pressure-bearing capacity, and tunable electrical resistance [7,23].

Due to poor natural convection heat transfer between the heating element and air, exposed elements experience significant temperature increase when energized. This results in higher temperatures at the sheet edges than in the central region, leading to localized overheating, joint deformation, and reduced strength—a phenomenon commonly termed as edge effect [20]. Research indicates that the edge effect can be effectively mitigated by applying pulsed or ramped voltage profiles [24], adjusting welding power parameters [25,26], actively cooling sheet edges [25], adjusting clamping distances [27], modifying heating element surface properties [27], reducing heated length of elements in the weld zone [28], and introducing ultrasonic assistance [29,30]. Furthermore, when the heating element contacts conductive fibers within the composite, unintended conductive pathways may form, causing current shunting. This reduces current density in the heating element [31] and compromises interfacial temperature uniformity [24,27]. The addition of pure resin layers [31], metal oxide coatings [27], and gradient pressure [32] on both sides of the heating element can effectively block the shunt path and enhance the interfacial bonding strength.

Continuous carbon fiber-reinforced polycarbonate composites (CCF/PC) have been widely used in the field of medical devices, automotive, and aerospace owing to their exceptional mechanical properties and processing characteristics. Nevertheless, research on welding techniques for CCF/PC—particularly resistance welding—remains limited. This study employs SS mesh as the heating element and incorporates pure PC resin interlayers on both sides. Through the development of an electro-thermal coupled finite element model in ABAQUS, we systematically investigated the effects of SS mesh structural parameters (aperture geometry, opening size, sheet thickness) and clamping distance on the temperature distribution and joint tensile-shear performance in CCF/PC resistance welding. Guided by simulations, a graded-aperture SS mesh was designed to optimize the thermal distribution on the welding interface. This work aims to provide both theoretical foundations and practical guidance for resistance welding of CCF/PC composites.

## 2. Materials and Methods

### 2.1. Materials

**Figure 1:**
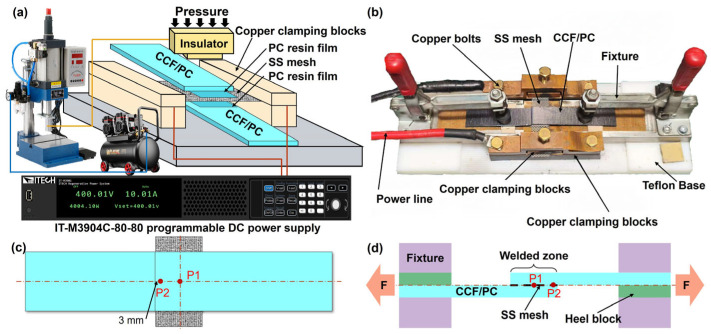
(**a**) Photograph of the self-designed resistance welding fixture; (**b**) schematic diagram of resistance welding system; (**c**) K-type thermocouple temperature measurement position diagram; (**d**) Schematic diagram of lap shear test.

CCF/PC sheets with dimensions of 100 mm × 25 mm × 2 mm were used as the experimental material (supplied by Nanjing Advanced Thermoplastic Composite Co., Ltd., Nanjing, China). A Type 304 stainless steel perforated mesh (SS mesh, 80 mm × 20 mm) was used as the heating element (supplied by Weifang Ruida Screen Co., Ltd., Weifang, China). Pure PC resin films (25 mm × 20 mm × 0.2 mm) were placed on both sides of the SS mesh (Figure 1a) to enhance interfacial bonding performance and prevent current shunting caused by direct contact between the SS mesh and carbon fibers in the sheets.

### 2.2. Welding Equipment and Process

As shown in Figure 1a,b, two CCF/PC sheets were fixed in the welding fixture using clamps. Both ends of the SS mesh were secured in copper clamping blocks and compressed with M10 bolts to ensure good electrical contact. An IT-M3904C-80-80 programmable DC power supply (ITECH Electronics, Nanjing, China) was used as the welding power source. The entire welding assembly was positioned under a TLQD pneumatic press connected to an air compressor (supplied by Changzhou Wanhe Electromechanical Co., Ltd., Changzhou, China), enabling precise control of welding pressure.

To prevent moisture vaporization from compromising joint quality, all CCF/PC sheets and PC resin films were dried at 120 °C and 100 °C, respectively, for 2 h. K-type thermocouples were positioned at the weld zone center (P1) and edge (P2) for real-time temperature monitoring (Figure 1c,d). Four specimens were welded per parameter set: three for lap-shear testing and one for cross-sectional analysis.

Lap-shear testing was performed using an ETM105D universal testing machine (produced by Shenzhen Wance Testing Machine Co., Ltd., Shenzhen, China) at 2 mm/min crosshead speed (Figure 1d).

Metallographic samples were ground stepwise using 240–2000 grit SiC paper, followed by polishing on a KMP-2DT polishing machine and ultrasonic cleaning in ethanol. Macroscopic features were examined using a Smart-Zoom 5 digital microscope (produced by Carl Zeiss Suzhou Co., Ltd., Suzhou, China), while microstructural characteristics were analyzed using a Zeiss Axio Vert.A1 optical microscope (produced by Carl Zeiss Suzhou Co., Ltd., Suzhou, China).

### 2.3. Finite Element Simulation

**Figure 2:**
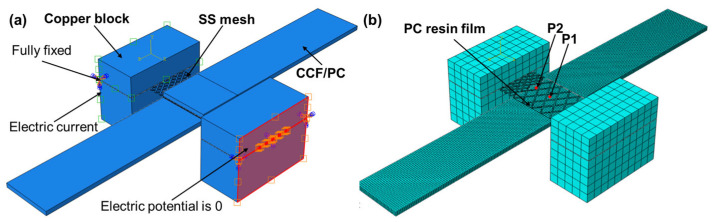
Resistance welding finite element model: (**a**) assembly drawing and boundary conditions; (**b**) mesh division diagram.

Finite element simulations of the resistance welding temperature field were performed using ABAQUS 2020 software. The model comprised copper clamps, SS mesh, PC resin films, and CCF/PC sheets, with material parameters detailed in Table 1, Table 2 and Table 3. The geometry model and boundary conditions are illustrated in Figure 2a. The specimen is fully fixed (i.e., all degrees of freedom constrained) at the clamping regions. One edge of the stainless steel (SS) mesh is grounded (electric potential = 0 V), while a surface current density corresponding to the designated welding current is applied to the opposite edge. A coupled electrothermal analysis was performed using DC3D8E elements (eight-node linear coupled thermo-electric hexahedral elements). Mesh information for each component is presented in Figure 2b and Table 4. The transient evolution of the temperature field during the CCF/PC resistance welding process was studied by defining two analysis steps: welding heating and holding-pressure cooling.

**Figure 3:**
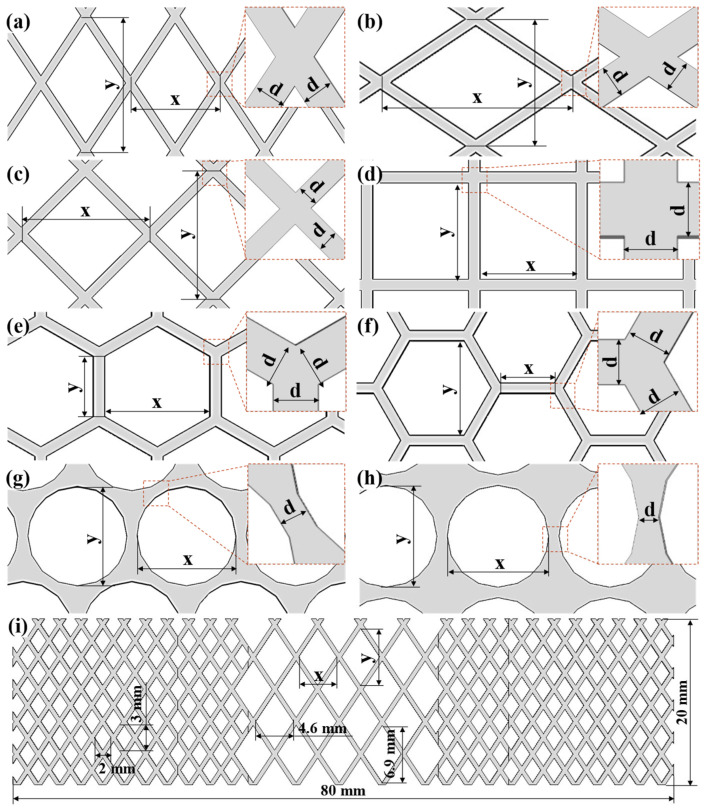
Schematic illustrations of SS meshes with different aperture shapes: (**a**) Diamond aperture (short diagonal parallel to current); (**b**) Diamond aperture (long diagonal parallel to current); (**c**) Square aperture (diagonal parallel to current); (**d**) Square aperture (non-oriented); (**e**) Hexagonal aperture (edge perpendicular to current); (**f**) Hexagonal aperture (edge parallel to current); (**g**) Circular aperture (hexagonal arrangement); (**h**) Circular aperture (square arrangement); (**i**) Novel mesh heating element with varying aperture size.

The aperture shapes of the SS mesh heating elements used in the simulations and experiments are shown in Figure 3. Detailed dimensions for the different aperture geometries and their corresponding welding process parameters are provided in Table 5. Specifically, Nos. 1, 2, and 3 were used to validate the accuracy of the finite element model and to analyze the influence of SS mesh thickness on the temperature field. Nos. 1 and 4–10 were used to investigate the effects of aperture shape and arrangement under identical aperture area (3 mm^2^) and aperture spacing (0.2 mm). Nos. 1 and 11–16 were used to study the combined influence of SS mesh thickness and aperture area, with aperture shape and arrangement held constant. Additionally, No. 17 represents a novel heating element structure designed in this study. The parameters used for all simulations (18 A welding current, 0.3 MPa welding pressure, 40 s welding time, and 40 s holding time) were kept consistent to compare the effect of geometric factor of the SS mesh heating elements on the welding process.

When defining the electric field boundary conditions, the right copper clamp block was assigned zero potential, while a current load corresponding to the welding current was applied to the left copper clamp block. Initial ambient temperature was set at 20 °C. All components were subjected to fixed constraints due to negligible displacement during the actual welding process. The indoor natural convection film heat transfer coefficient is uniformly taken as 7 W/(m^2^·K). The emissivities of the SS mesh, copper electrode, and CF/PC are 0.2, 0.03, and 0.9, respectively. Heat convection and thermal radiation can be expressed by Equation (1) and Equation (2), respectively.

(1) $$-k\nabla T\cdot n=h(T-T_{\infty })$$

(2) $$-k\nabla T\cdot n=\varepsilon \sigma_{SB}(T^{4}-T_{\infty }^{4})$$

In Equation (1), *h* denotes the convective heat transfer coefficient (W/(m^2^·K)), which is typically set in the range of 5–25 W/(m^2^·K) for natural convection; *T*_∞_ is the ambient temperature (K); and **n** is the outward unit normal vector on the surface. In Equation (2), εrepresents the surface emissivity, and *σ*_SB_ is the Stefan–Boltzmann constant, with a value of 5.67 × 10^−8^ W/(m^2^·K^4^).

Contact pairs were defined with the copper clamp as the master surface against the SS mesh, the SS mesh as the master surface against the PC film, and the PC film as the master surface against the CCF/PC sheet. The interfacial thermal conductance coefficients at the three contact interfaces were set as 56,000 W/(m^2^·K), 1000 W/(m^2^·K), and 1200 W/(m^2^·K), respectively. Their corresponding electrical conductivities are 2,762,430 S/m, 0 S/m, and 0 S/m, respectively [33]. Contact properties included tangential behavior, normal behavior, thermal conductance, heat generation effects, and electrical conductance. All contact interfaces were assumed to maintain effective electrical and thermal contact under sufficient applied pressure. Electrical conduction, transient heat conduction, and interfacial thermal contact conductance can be described by Equations (3), (4), and (5), respectively.

(3) ∇⋅(σ∇ϕ)=0

(4) $$\rho c_{p}\frac{\partial T}{\partial t}=\nabla \cdot (k\nabla T)+Q_{Joule}$$

(5) $$-k_{1}\nabla T_{1}\cdot n_{1}=-k_{2}\nabla T_{2}\cdot n_{2}=h_{c}(T_{1}-T_{2})$$

In Equation (3), *σ* denotes the electrical conductivity of the material, and *ϕ* represents the electric potential. In Equation (4), *T*, *ρ*, and *C*_P_ denote temperature, density, and specific heat capacity, respectively; k is the thermal conductivity; and *Q*_Joule_ is the Joule heating source term. In Equation (5), *R*_c_ represents the thermal contact resistance; *h*_c_ is the interfacial thermal conductance; and *T*_1_ and *T*_2_ are the temperatures on the two sides of the interface, respectively.

## 3. Results and Discussion

### 3.1. Validation of Finite Element Model

**Figure 4:**
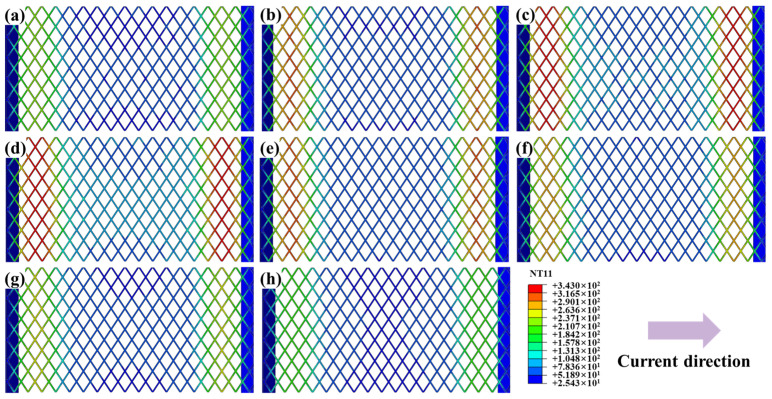
Temperature field distribution for the No.2 SS mesh at varying time intervals: (**a**) 10 s; (**b**) 20 s; (**c**) 30 s; (**d**) 40 s; (**e**) 50 s; (**f**) 60 s; (**g**) 70 s; (**h**) 80 s.

To validate the accuracy of the finite element model, the simulated and measured temperature using the Nos.1, 2, and 3 SS meshes were compared. The temperature field of the No. 1 SS mesh at different times is shown in Figure 4. To clearly present the surface temperature of the SS mesh, the upper and lower sheets as well as the pure PC films were hidden. It can be observed that during the 0–40 s resistive heating process (Figure 4a–d), a continuous increase in surface temperature occurs. The regions near the copper clamps exhibit lower temperatures due to rapid heat dissipation through conduction, while the central region shows a faster heating rate, and the high-temperature zone gradually expands toward the edges. Throughout the 40–80 s pressure-holding cooling phase (Figure 4e–h), the central region exhibited delayed temperature decay in the central region due to thermal inertia and limited clamp contact, contrasting with rapid cooling in peripheral regions through efficient heat transfer to copper clamps.

**Figure 5:**
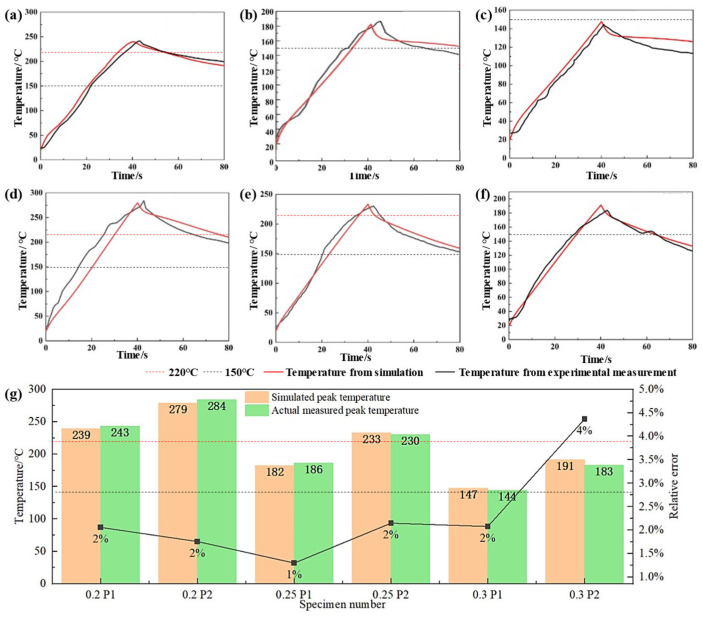
Comparison of simulated and experimentally measured temperatures for SS meshes with varying thicknesses: (**a**) 0.2 mm at P1; (**b**) 0.25 mm at P1; (**c**) 0.3 mm at P1; (**d**) 0.2 mm at P2; (**e**) 0.25 mm at P2; (**f**) 0.3 mm at P2; (**g**) Peak temperature comparison.

Figure 5 compares simulated and measured temperatures during resistance welding of SS meshes with varying thicknesses. For identical thicknesses, the edge node P2 consistently exhibited higher temperatures than the central node P1 at all timepoints. Increasing SS mesh thickness reduced overall electrical resistance, thereby lowering peak surface temperatures. Unlike thermocouple measurements affected by environmental perturbations (e.g., thermal response lag, contact resistance fluctuations, convective variations), simulated curves demonstrated greater stability. While minor discrepancies existed in heating rates and near-peak regions, simulations showed strong agreement with experimental data in temperature evolution trends, heating phases, peak temperature magnitudes, and cooling profiles. Relative errors between simulated and experimental peak temperatures ranged from 1.30% to 4.37% (Figure 5g), confirming excellent model accuracy and reliability. This validated model effectively predicts heat generation and temperature distribution in SS meshes with diverse structural parameters, thus providing a robust foundation for subsequent process optimization and structural design.

### 3.2. Influence of SS Mesh Geometric Parameters on Temperature Field

**Figure 6:**
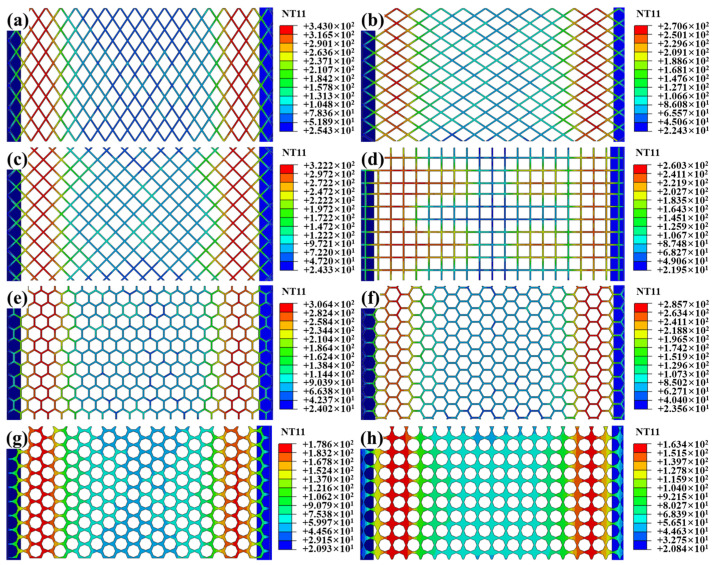
Temperature fields of SS meshes with distinct aperture geometries: (**a**) SS mesh with No.1; (**b**) SS mesh with No.4; (**c**) SS mesh with No.5; (**d**) SS mesh with No.6; (**e**) SS mesh with No.7; (**f**) SS mesh with No.8; (**g**) SS mesh with No.9; (**h**) SS mesh with No.10.

SS meshes Nos. 1 and 4–10 were selected to investigate the influence of aperture geometry on heating performance, with results presented in Figure 6. To better display the surface temperature of the SS mesh, the sheets and the pure PC film are also hidden. The peak temperature decreases in the following order: Peak temperatures decreased in the following order: diamond apertures (short diagonal parallel to current) (Figure 6a), square apertures (diagonal parallel to current) (Figure 6c), hexagonal apertures (edge perpendicular to current) (Figure 6e), hexagonal apertures (edge parallel to current) (Figure 6f), diamond apertures (long diagonal parallel to current) (Figure 6b), square apertures (non-oriented) (Figure 6d), circular apertures (hexagonal arrangement) (Figure 6g), and circular apertures (square arrangement) (Figure 6h)—thus directly reflects decreasing equivalent electrical resistance under identical aperture area constraints.

The equivalent resistance of a SS mesh can be expressed by Equation:

(6) $$R=\rho \frac{L_{eff}}{A_{eff}}$$

where ρ is the resistivity (material-dependent), *L_eff_* is the effective current path length, and *A_eff_* is the effective cross-sectional area perpendicular to current flow. Aperture geometry modulates resistance primarily through *L*_eff_ and *A*_eff_, with shorter flow-direction projected length (FPL) reducing *A*_eff_ and increased path tortuosity elongating *L*_eff_. Combined analysis of Table 6 and Equation (6) establishes a definitive resistance hierarchy. Diamond apertures with minor axis aligned parallel to current flow exhibit maximal resistance due to extreme path tortuosity along the major axis, maximizing *L*_eff_. Concurrently, their minimal FPL minimizes *A*_eff_. Square apertures with their diagonal oriented parallel to the current exhibit the second-highest resistance. This configuration yields moderate tortuosity and intermediate *A*_eff_ values. Hexagonal apertures demonstrate lower resistance when edges are parallel to current flow. Conversely, transverse edge orientation increases resistance through reduced FPL. Diamond apertures with major axis parallel to current achieve near-minimal resistance. Their near-linear current paths drastically reduce *L*_eff_. Non-oriented squares further decrease resistance via reduced average tortuosity. Circular apertures deliver the lowest resistance owing to uniform current distribution and high conductive area ratio. Notably, hexagonal packing elevates resistance versus square packing. This occurs through higher areal density reducing *A*_eff_. This hierarchy confirms diamond-minor-axis alignment as optimal for high-heat applications. Meanwhile, circular-square packing maximizes conductivity for uniform heating requirements.

**Figure 7:**
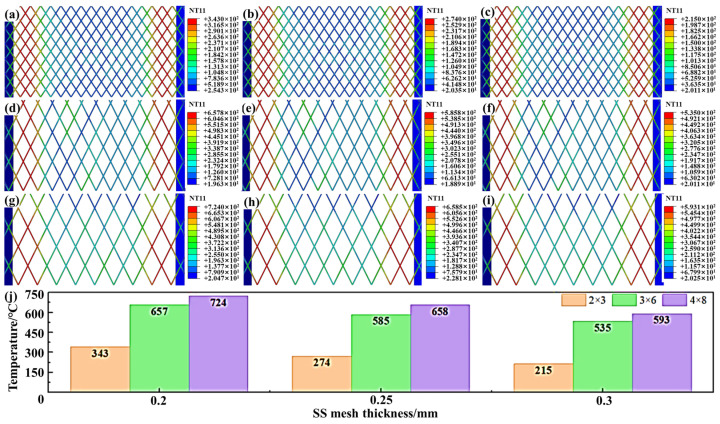
Temperature field comparisons for SS meshes with varying dimensions: (**a**) No.1 SS mesh; (**b**) No.2 SS mesh; (**c**) No.3 SS mesh; (**d**) No.11 SS mesh; (**e**) No.12 SS mesh; (**f**) No.13 SS mesh; (**g**) No.14 SS mesh; (**h**) No.15 SS mesh; (**i**) No.16 SS mesh; (**j**) Peak temperature comparison.

SS meshes Nos. 1 and 11–16 were selected to investigate the effects of aperture area and mesh thickness on heating performance, and the results are presented in Figure 7. It can be observed that, at identical aperture areas, increasing the thickness reduces resistance due to increased *A*_eff_, lowering peak temperatures. Conversely, increasing aperture size at constant thickness increases the *L*_eff_ and reduces the *A*_eff_, and thereby increases overall resistance. This leads to increased Joule heating for the same current input, substantially raising peak temperatures. Thus, aperture size plays a critical role in determining thermal generation efficiency.

Building on the established electrothermal relationships, we deduce that for SS meshes with identical thickness, aperture area, and wire spacing, diamond apertures exhibit increasing resistance with greater aspect ratio (i.e., higher major-to-minor axis ratio) when the minor axis is aligned parallel to the current direction. This configuration maximizes current path tortuosity (*L_eff_*) while minimizing the effective cross-sectional area (*A_eff_*), thereby maximizing electrical resistance. Given the superior heating performance of this configuration, it is adopted as the standard geometry and orientation for all subsequent simulations and experiments.

### 3.3. Influence of Clamping Distance on Welding Temperature Field

To investigate the effect of clamping distance on temperature distribution, SS mesh No. 1 was selected, and the distance between the copper blocks and the CCF/PC sheets was set at 8.5 mm, 5 mm, and 1 mm.

**Figure 8:**
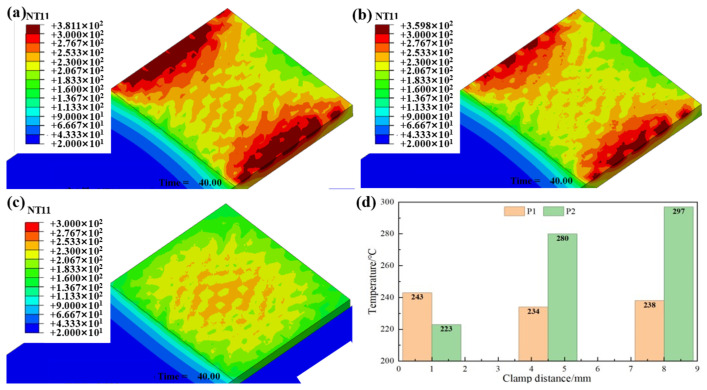
Temperature field distribution on workpiece surfaces at varying clamping distances: (**a**) 8.5 mm; (**b**) 5 mm; (**c**) 1 mm; (**d**) Peak temperature comparison.

As shown in Figure 8a, when the clamping distance was 8.5 mm, obvious edge effect, i.e., the temperature and the edges of the overlap region is higher than the center region (~50 °C), can be observed. This is due to the inefficient natural convection between the heating element and the surrounding air, causing overheating in the exposed regions of the heating element. This could lead to the localized overheating at the joint edges, potentially degrading the polymer matrix and compromising the joint integrity. Reducing clamping distance to 5 mm (Figure 8b) and 1 mm (Figure 8c) progressively reduced peripheral temperatures while improving overall uniformity. At a 1 mm clamping distance, central temperatures were ~20 °C higher than those in peripheral regions, effectively mitigating edge overheating. These results demonstrate that minimizing clamping distance significantly enhances temperature homogeneity within the weld zone, thereby preventing material degradation caused by localized thermal extremes.

### 3.4. Influence of SS Mesh Thickness on Joint Performance

In CCF/PC resistance welding, the interfacial temperature distribution critically determines joint quality. When temperatures exceed the glass transition temperature of the PC matrix (T_g_~150 °C), the resin softens and becomes flowable. Above the viscous flow temperature (~220 °C), polymer chain mobility increases substantially, enabling molecular interdiffusion and entanglement at the interface to form robust welded joints.

Under welding parameters of 18 A current, 0.3 MPa pressure, 40 s welding time, and 40 s holding time, Figure 5 and Table 7 illustrated the temperature evolution and mechanical properties of joints fabricated with SS meshes of varying thicknesses: the 0.2-mm-thick SS mesh exhibited higher electrical resistance, leading to significant heat generation. Peak temperatures at points P1 and P2 reached 243 °C and 284 °C, respectively. Both locations sustained temperatures above 220 °C for over 20 s, which was sufficient to ensure complete resin melting and molecular chain diffusion. This resulted in excellent interfacial bonding after cooling, with a maximum lap-shear force of 8.713 kN. As the thickness increased to 0.25 mm, the larger cross-sectional area reduced electrical resistance and heat generation, resulting in peak temperatures of 186 °C at P1 and 230 °C at P2. The central region did not reach the flow temperature (~220 °C), whereas the peripheral region briefly entered the flow state with limited melting. This led to inadequate resin impregnation into the SS mesh and weakened interfacial bonding, reducing the maximum lap-shear force to 2.383 kN. Further increasing the thickness to 0.3 mm significantly reduced heating efficiency, resulting in peak temperatures of 144 °C at P1 and 183 °C at P2—both below the PC’s T_g_ (~150 °C) and flow temperature. Consequently, the resin remained solid without softening or flow, thereby preventing the formation of an effective weld.

**Figure 9:**
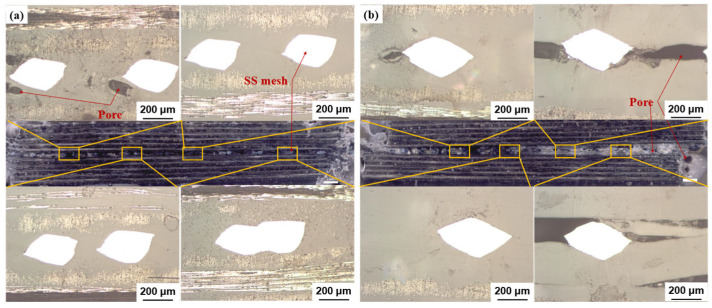
Cross-sectional macro- and micro-morphologies of welded joints: (**a**) SS mesh with No.1; (**b**) SS mesh with No.2. *Note: White regions denote SS wires; black areas represent pores*.

Figure 9 shows the macro- and micro-cross-sectional morphologies of joints fabricated with SS meshes with varying thicknesses. At 0.2 mm thickness, full melting of the PC resin enabled thorough encapsulation of SS wires, yielding dense interfacial bonding with minimal pores and excellent mechanical property. At 0.25 mm thickness, substantial porosity and unmelted zones resulted from inadequate resin impregnation into SS mesh, causing interfacial debonding and weakening defects [24]. These findings demonstrate that SS mesh thickness critically influences electrical resistance and heat generation, thereby determining the thermal input at the welding interface and ultimately governing joint quality. The optimal parameters identified for 0.2-mm SS meshes are not unsuitable for thicker variants, underscoring the necessity of matching welding parameters to heating element dimensions to achieve high-quality, reliable resistance welds.

### 3.5. Novel Mesh Heating Element with Varying Aperture Size

As stated in Figure 7, a larger aperture contributes to higher temperature. This study proposed an innovative SS mesh structure with varying aperture size (Figure 3i) to mitigate the edge effect and improve interfacial temperature uniformity. The design incorporates enlarged apertures (4.6 mm × 6.9 mm) within the weld zone to reduce A_eff_, thereby increasing local electrical resistance and heat generation. Peripheral regions employ smaller apertures (2 mm × 3 mm) to maintain higher conductive cross-sectional area, reducing resistance and heat output. This configuration ensures sufficient heating at the center while suppressing excessive edge temperatures, effectively avoiding SS mesh burnout and matrix degradation caused by peripheral overheating.

**Figure 10:**
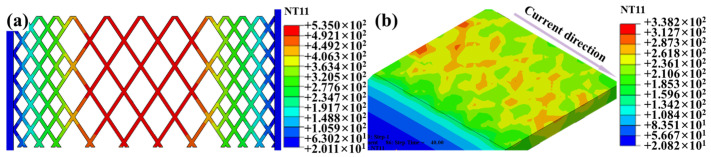
Welding temperature field at a welding time of 40 s: (**a**) SS mesh; (**b**) welding interface.

Finite element simulation was conducted on this novel SS mesh, and the results are shown in Figure 10. The clamping distance was set as 8.5 mm in the simulation. It can be seen that the temperature distribution becomes more uniform on the welding interface than the case of using conventional SS mesh (as shown in Figure 8). The temperature difference between the edge (P2) and center (P1) was 30 °C.

Welding trials were conducted to preliminary investigate the effect of this novel mesh heating element, and the results are listed in Table 8. It can be seen that the optimal lap shear force reached 9.851 kN (13.1% enhancement over conventional uniform-aperture structures, 8.713 kN) when the welding parameters was 14 A welding current, 40 s welding time, 40 s holding time, and 0.3 MPa welding pressure.

**Figure 11:**
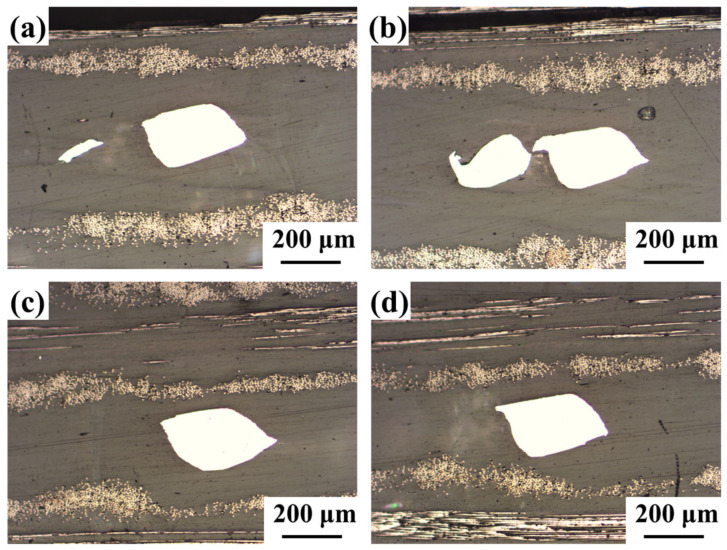
Cross-sectional morphology of the joint: (**a**,**b**) edge region; (**c**,**d**) center region.

Figure 11 shows the corresponding cross-sectional morphology. Both central and edge regions show complete resin infiltration throughout the SS mesh interstices, with no observable pores, lack of fusion, or interfacial debonding, suggesting uniform temperature distribution and excellent process stability [31].

## 4. Conclusions

This study investigated the effects of SS mesh structural parameters (aperture geometry, opening area, thickness) and fixture parameters (clamping distance) on the temperature field and joint performance of CCF/PC resistance welding by employing a coupled electro-thermal finite element model. The main conclusions are given as follows.

(1) The developed finite element model accurately captured the temperature evolution during the welding process, with a relative error between simulated and experimentally measured temperatures of only 1–4%.
(2) Under identical conditions of thickness, aperture spacing, and opening area ratio, SS meshes with rhombic apertures—oriented such that the short diagonal is aligned parallel to the current direction—exhibited higher heating efficiency. Moreover, the electrical resistance of the SS mesh increased with decreasing thickness and increasing open area ratio.
(3) Reducing the clamping distance improved the temperature uniformity across the lap zone, mitigated the edge effect, and effectively suppressed resin degradation near the specimen edges, thereby enhancing interfacial bonding at the joint.
(4) The SS mesh with a thickness of 0.2 mm and diamond apertures measuring 2 mm × 3 mm demonstrated the highest heating efficiency. Experimental optimization identified the optimal welding parameters as: 18 A current, 40 s welding time, 40 s holding time, and 0.3 MPa clamping pressure, achieving a tensile shear force of 8.713 kN.
(5) A novel SS mesh design—featuring larger openings in the weld zone to enhance local heating and smaller peripheral openings to suppress edge effect—improved temperature uniformity and yielded a tensile shear force of 9.851 kN, with defect-free cross-sectional morphology observed.

## Figures and Tables

**Table 1:** Material parameters of copper clamps.

Temperature/°C	Coefficient of Thermal Expansion/K^−1^ × 10^−6^	Elasticity Modulus/GPa	Density/kg·m^−3^	Electrical Resistivity/Ω·m × 10^−8^	Thermal Conductivity/W·m^−1^·K^−1^	Specific Heat/J·kg^−1^·K^−1^
20	16.6	124	8747	2.64	390.62	397.75
100	16.7	105	8747	3.00	370.42	401.93
200	17.1	93.0	8747	3.99	355.45	418.68
300	17.5	82.7	8747	5.05	345.72	431.24
500	18.4	38.6	8747	6.99	320.28	452.17
600	18.5	24.8	8747	8.00	315.79	464.73
800	19.3	13.8	8747	9.48	305.32	477.30

**Table 2:** Material parameters of SS mesh heating element.

Temperature/°C	Coefficient of Thermal Expansion/K^−1^ × 10^−6^	Elasticity Modulus/GPa	Density/kg·m^−3^	Electrical Resistivity/Ω·m × 10^−8^	Thermal Conductivity/W·m^−1^·K^−1^	Specific Heat/J·kg^−1^·K^−1^
20	17.0	198	7930	73	14.6	462
100	17.4	193	7880	86	15.1	496
200	18.0	185	7830	106	16.1	512
300	18.6	176	7790	121	17.9	525
500	19.1	167	7750	121	18.0	540
600	19.6	159	7660	121	20.8	577
800	20.2	151	7560	121	23.9	604
1200	20.7	60	7370	130	32.2	676
1400	21.1	20	7320	161	33.7	692
1500	21.6	10	7320	170	120.0	700

**Table 3:** Material parameters of PC resin film and CCF/PC sheets.

Material	Coefficient of Thermal Expansion/K^−1^ × 10^−6^	Elasticity Modulus/GPa	Density/kg·m^−3^	Electrical Resistivity/Ω·m × 10^−8^	Specific Heat/J·kg^−1^·K^−1^
PC	68	2.3	1200	0.220	1200
CCF/PC	—	4.0	1350	0.763	1735

**Table 4:** Dimensions and mesh information of each component.

Component	Length/mm	Width/mm	Thickness/mm	Number of Nodes	Number of Elements
Top copper block	20	40	10	150	264
SS mesh	80	20	-	-	-
Bottom copper block	20	40	20	250	396
PC Film	20	25	0.2	2850	5916
CCF/PC sheet	100	25	2.0	71,250	87,348

**Table 5:** Thickness and mesh information of all SS meshes.

No.	Thickness/mm	Aperture Shape	Aperture Spacing d/mm	Dimensions of x × y/mm × mm	Aperture Area/mm^2^	Number of Elements	Number of Nodes
1	0.2	a	0.2	2 × 3	3	1457	3802
2	0.25	a	0.2	2 × 3	3	2484	5181
3	0.3	a	0.2	2 × 3	3	2568	5732
4	0.2	b	0.2	3 × 2	3	1711	5678
5	0.2	c	0.2	2.449 × 2.449	3	1535	4206
6	0.2	d	0.2	1.732 × 1.732	3	1321	3848
7	0.2	e	0.2	2.148 × 1.074	3	2609	7234
8	0.2	f	0.2	1.074 × 2.148	3	2723	7576
9	0.2	g	0.2	1.954 × 1.954	3	3143	9902
10	0.2	h	0.2	1.954 × 1.954	3	2658	6890
11	0.2	a	0.2	3 × 6	9	1914	4263
12	0.25	a	0.2	3 × 6	9	1936	4296
13	0.3	a	0.2	3 × 6	9	1994	4383
14	0.2	a	0.2	4 × 8	16	964	3172
15	0.25	a	0.2	4 × 8	16	1086	3279
16	0.3	a	0.2	4 × 8	16	1293	3348
17	0.2	i	0.2	2 × 3, 4.6 × 6.9	6, 15.87	1945	5824

**Table 6:** Thermal and dimensional characteristics of SS meshes with different aperture shapes.

No.	Aperture Shape	Peak Temp./°C	Min. Temp./°C	FPL ^1^/mm	Current Path Length/mm	Conductive Area Fraction	Simulated Resistance/mΩ
1	Diamond	343.0	25.43	2.000	3.605	20.30%	217.5
5	Square	322.2	24.33	2.449	3.964	19.61%	189.6
7	Hexagon	306.4	24.02	1.862	3.225	18.48%	172.1
8	Hexagon	285.7	23.56	2.150	3.225	18.48%	159.8
4	Diamond	270.6	22.43	3.000	3.605	20.30%	133.1
6	Square	260.3	21.95	1.732	1.732	19.61%	126.7
9	Circular	178.6	20.93	1.954	3.070	25.38%	79.42
10	Circular	163.4	20.84	1.954	3.070	35.37%	74.67

^1^ FPL: Flow-direction Projected Length.

**Table 7:** Temperature profiles and maximum lap-shear force for SS meshes of varying thicknesses.

No.	SS Mesh Thickness/mm	Peak Temp. at P1/°C	Peak Temp. at P2/°C	Duration > 150 °C at P1/s	Duration > 220 °C at P1/s	Duration > 150 °C at P2/s	Duration > 220 °C at P2/s	Max. Lap-Shear Force/kN
1	0.2	243	284	39	20	65	37	8.713
2	0.25	186	230	35	0	60	9	2.383
3	0.3	144	183	0	0	35	0	0

Note: P1/P2 denote thermocouple positions at edge/center of weld zone (Figure 1c,d).

**Table 8:** Resistance welding test data of using heating element with varying aperture size.

No.	Welding Current /A	Welding Time /s	Holding Time /s	Welding Pressure /MPa	Average Lap Shear Force /kN
1	18	40	40	0.3	8.264
2	18	35	40	0.3	9.415
3	18	37	40	0.3	8.159
4	18	33	40	0.3	6.443
5	16	35	40	0.3	8.447
6	15	35	40	0.3	9.438
7	14	35	40	0.3	8.936
8	15	42	40	0.3	7.463
9	14	40	40	0.3	9.851

## Data Availability

The data presented in this study are available upon request from the corresponding author.
